# Triaqua­(benzene-1,3-dicarboxyl­ato)(4,5-diaza­fluoren-9-one)cadmium(II) penta­hydrate

**DOI:** 10.1107/S1600536810045551

**Published:** 2010-11-13

**Authors:** Wei Fang

**Affiliations:** a School of Chemical Engineering & Technology, Harbin Institute of Technology, Harbin 150001, People’s Republic of China, and Department of Chemistry, Baicheng Normal University, Baicheng 137000, People’s Republic of China

## Abstract

In the title compound, [Cd(C_8_H_4_O_4_)(C_11_H_6_N_2_O)(H_2_O)_3_]·5H_2_O, the Cd^II^ atom is seven-coordinated by two N atoms from one bidentate phenanthroline-derived ligand and by five O atoms, two from one bidentate benzene-1,3-dicarboxyl­ate (1,3-BDC) ligand and three from water mol­ecules, in a distorted penta­gonal-bipyramidal geometry. Neighbouring units inter­act through π–π inter­actions [centroid–centroid distances = 3.380 (3) and 3.283 (4) Å]. Finally, three types of O—H⋯O hydrogen bonds exist between coordinated dissociative water mol­ecules and hybridization water mol­ecules and carboxyl­ate O atoms, resulting in a two-dimensional network parallel to (010).

## Related literature

For applications of 1,10-phenanthroline and its derivatives in the construction of metal-organic complexes, see: Li *et al.* (2006 ▶, 2009 ▶); Olivier *et al.* (2008 ▶); Hong *et al.* (2009 ▶). For π–π stacking in related structures, see: Noveron *et al.* (2002 ▶). For the synthesis of 4,5-diaza­fluorene-9-one, see: Henderson *et al.* (1984 ▶).
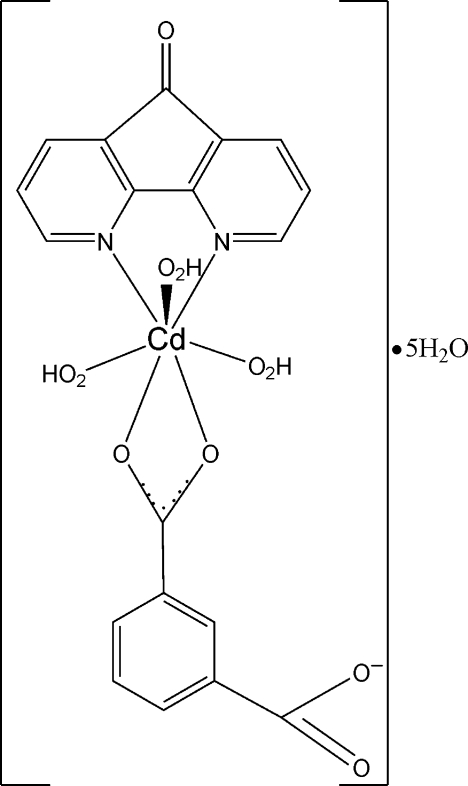


## Experimental

### 

#### Crystal data

[Cd(C_8_H_4_O_4_)(C_11_H_6_N_2_O)(H_2_O)_3_]·5H_2_O
*M* *_r_* = 602.83
Monoclinic, 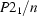

*a* = 7.1218 (6) Å
*b* = 31.893 (3) Å
*c* = 11.0278 (9) Å
β = 106.579 (1)°
*V* = 2400.7 (4) Å^3^
*Z* = 4
Mo *K*α radiation
μ = 0.98 mm^−1^
*T* = 292 K
0.54 × 0.23 × 0.18 mm

#### Data collection

Bruker SMART diffractometer
Absorption correction: multi-scan (*SADABS*; Bruker, 1998 ▶) *T*_min_ = 0.762, *T*_max_ = 0.839
14040 measured reflections
4721 independent reflections
3839 reflections with *I* > 2σ(*I*)
*R*_int_ = 0.064

#### Refinement

*R*[*F* ^2^ > 2σ(*F* ^2^)] = 0.051
*wR*(*F* ^2^) = 0.119
*S* = 1.07
4721 reflections
325 parameters
H-atom parameters constrained
Δρ_max_ = 0.85 e Å^−3^
Δρ_min_ = −0.81 e Å^−3^

### 

Data collection: *SMART* (Bruker, 1998 ▶); cell refinement: *SAINT* (Bruker, 1998 ▶); data reduction: *SAINT*; program(s) used to solve structure: *SHELXS97* (Sheldrick, 2008 ▶); program(s) used to refine structure: *SHELXL97* (Sheldrick, 2008 ▶); molecular graphics: *SHELXTL* (Sheldrick, 2008 ▶); software used to prepare material for publication: *SHELXTL*.

## Supplementary Material

Crystal structure: contains datablocks global, I. DOI: 10.1107/S1600536810045551/nk2061sup1.cif

Structure factors: contains datablocks I. DOI: 10.1107/S1600536810045551/nk2061Isup2.hkl

Additional supplementary materials:  crystallographic information; 3D view; checkCIF report

## Figures and Tables

**Table 1:** Hydrogen-bond geometry (Å, °)

*D*—H⋯*A*	*D*—H	H⋯*A*	*D*⋯*A*	*D*—H⋯*A*
O*W*1—H*W*1*A*⋯O4^i^	0.84	1.82	2.660 (4)	177
O*W*1—H*W*1*B*⋯O*W*4^ii^	0.84	1.88	2.705 (5)	168
O*W*2—H*W*2*A*⋯O4^iii^	0.84	1.95	2.700 (5)	148
O*W*3—H*W*3*B*⋯O*W*8^iv^	0.84	2.11	2.945 (6)	174
O*W*3—H*W*3*A*⋯O*W*7	0.89	1.93	2.756 (5)	154
O*W*5—H*W*5*A*⋯O*W*7^v^	0.85	1.93	2.714 (6)	154
O*W*4—H*W*4*B*⋯O2	0.84	1.96	2.787 (5)	167
O*W*6—H*W*6*A*⋯O3^vi^	0.84	1.90	2.725 (5)	166
O*W*7—H*W*7*B*⋯O*W*6^vii^	0.84	1.91	2.715 (5)	159
O*W*8—H*W*8*A*⋯O3^vi^	0.84	1.92	2.717 (5)	158
O*W*8—H*W*8*A*⋯O4^vi^	0.84	2.52	3.230 (5)	143
O*W*8—H*W*8*B*⋯O*W*6^vii^	0.84	2.01	2.839 (6)	172
O*W*6—H*W*6*B*⋯O*W*3	0.83	2.37	3.079 (5)	144
O*W*6—H*W*6*B*⋯O*W*2	0.83	2.51	3.055 (6)	124
O*W*7—H*W*7*A*⋯O1	0.92	1.78	2.646 (5)	155

Symmetry codes: (i) 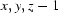; (ii) 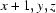; (iii) 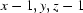; (iv) 
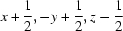
; (v) 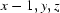; (vi) 
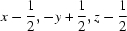
; (vii) 
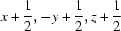
.

**Fig. 1.**
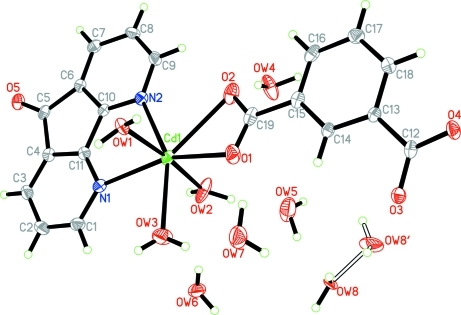
View of the local coordination of Cd(II) with the atomic numbering scheme. Displacement ellipsoids are drawn at the 30% probability level. (arbitrary spheres for the H atoms).

**Fig. 2.**
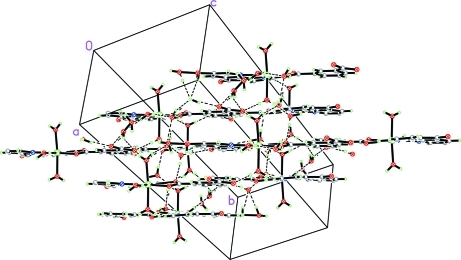
A view down crystallographic axis a of the two-dimensional supramolecular structure of (I) generated by π-π interaction and hydrogen-bonding.

## supplementary crystallographic information

### Comment

Over the past decade, coordination complexes in which rigid linear π-conjugated organic chains span transition metal centres have been proposed as models for molecular wires or for molecular (Olivier *et al.*, 2008). Organocopper, copper cadmium and zinc complexes have been widely explored due to their rich structural chemistry (Hong *et al.*, 2009). The chelating 1,10-phenanthroline (phen) and its derivatives are important ligands with numerous applications in the construction of metal-organic complexes(see, for example, Li *et al.*,2006). A our ongoing part studies in this area (Li *et al.*, 2009). Here, we reacted phen derivative 4,5-diazafluorene-9-one (C_11_H_6_N_2_O; *L*) with Cd^II^ and benzene-1,3-dicarboxylate (C_8_H_4_O_4_^2-^; 1,3-BDC), resulting in the title polymeric complex (I). In compound (I), the Cd^II^ atom of unit is surrounded by two N atoms derived from the bidentate *L* ligand, two O atoms from a bidentate 1,3-BDC ligand and thr ee O atoms from three H_2_O moleculars. The distances between Cd and O rang from 2.226 (4) to 2.644 (4). This results in a very distorted CdN_2_O_5_ pentagonal bipyramid with the donor atoms of both the bidentate species occupying both an equatorial and an axial site (Table 1, Fig.1). Neighbouring units in (I) are connected through π-π interactions between *L* ligands with π–π stacking distances of 3.380 (3) and 3.283 (4) Å. Similar values occur in related structures (Noveron *et al.*,2002). Finally, three types of hydrogen bonds exist between coordinated dissociative water molecules and hybridization water molecules and carboxylate group oxygen atoms. The related parameters are listed in (Table 2). resulting in a two-dimensional supramolecular structure. (Fig.2) A l l the H atoms carried on O atoms were located by Forrier map and then refined as riding atoms with *U*_iso_(H)= 1.2 times *U*_eq_(O). One of the water oxygen atoms is disordered in two positions as Ow8 and Ow8'. The occupancies of Ow8 and Ow8' were assigned as 0.75 and 1/4, respectively.

The structure has been refined to add an extra water molecule Ow8' in the vicinity of Ow8. Ow8 and Ow8' atoms have been considered as two disodered parts of one water oxygen atom. By this way, there were no any large residual peaks more than 1.0 e.A^-3^ in the final refinement.

### Experimental

Ligand was synthesized according to the literature method. (Henderson *et al.*, 1984). A mixture of CdCl_2_ (0.3 mmol), *L* (0.1 mmol) and H_2_1,3-BDC (0.3 mmol) in distilled water (30 ml) was stirred thoroughly for 1 h at ambient temperature. The pH was adjusted to 7.5 with aqueous NaOH solution. The suspension was then sealed in a Teflon-lined stainless steel reaction vessel (40 ml). The reaction was performed under autogeneous pressure and static conditions in an oven at 443 K for 4.5 d. The vessel was then cooled slowly inside the oven to 298 K at a rate of 5 K h^-1^ before opening: yellow crystals of (I) were collected.

### Refinement

All H atoms on C atoms were generated geometrically and refined as riding atoms with C—H = 0.93Å and *U*_iso_(H) = 1.2*U*_eq_(C).

### Crystal data

**Table:**

[Cd(C_8_H_4_O_4_)(C_11_H_6_N_2_O)(H_2_O)_3_]·5H_2_O	*F*(000) = 1224
*M**_r_* = 602.83	*D*_x_ = 1.665 Mg m^−^^3^
Monoclinic, *P*2_1_/*n*	Mo *K*α radiation, λ = 0.71073 Å
*a* = 7.1218 (6) Å	θ = 2.0–26.3°
*b* = 31.893 (3) Å	µ = 0.98 mm^−^^1^
*c* = 11.0278 (9) Å	*T* = 292 K
β = 106.579 (1)°	Block, yellow
*V* = 2400.7 (4) Å^3^	0.54 × 0.23 × 0.18 mm
*Z* = 4	

### Data collection

**Table:**

Bruker SMART diffractometer	4721 independent reflections
Radiation source: fine-focus sealed tube	3839 reflections with *I* > 2σ(*I*)
graphite	*R*_int_ = 0.064
Detector resolution: 0 pixels mm^-1^	θ_max_ = 26.0°, θ_min_ = 2.0°
ω scans	*h* = −5→8
Absorption correction: multi-scan (*SADABS*; Bruker, 1998)	*k* = −39→39
*T*_min_ = 0.762, *T*_max_ = 0.839	*l* = −13→13
14040 measured reflections	

### Refinement

**Table:**

Refinement on *F*^2^	Primary atom site location: structure-invariant direct methods
Least-squares matrix: full	Secondary atom site location: difference Fourier map
*R*[*F*^2^ > 2σ(*F*^2^)] = 0.051	Hydrogen site location: inferred from neighbouring sites
*wR*(*F*^2^) = 0.119	H-atom parameters constrained
*S* = 1.07	*w* = 1/[σ^2^(*F*_o_^2^) + (0.0547*P*)^2^ + 1.8475*P*] where *P* = (*F*_o_^2^ + 2*F*_c_^2^)/3
4721 reflections	(Δ/σ)_max_ = 0.001
325 parameters	Δρ_max_ = 0.85 e Å^−^^3^
0 restraints	Δρ_min_ = −0.81 e Å^−^^3^

### Special details

**Table:**

Geometry. All e.s.d.'s (except the e.s.d. in the dihedral angle between two l.s. planes) are estimated using the full covariance matrix. The cell e.s.d.'s are taken into account individually in the estimation of e.s.d.'s in distances, angles and torsion angles; correlations between e.s.d.'s in cell parameters are only used when they are defined by crystal symmetry. An approximate (isotropic) treatment of cell e.s.d.'s is used for estimating e.s.d.'s involving l.s. planes.
Refinement. Refinement of *F*^2^ against ALL reflections. The weighted *R*-factor *wR* and goodness of fit *S* are based on *F*^2^, conventional *R*-factors *R* are based on *F*, with *F* set to zero for negative *F*^2^. The threshold expression of *F*^2^ > σ(*F*^2^) is used only for calculating *R*-factors(gt) *etc*. and is not relevant to the choice of reflections for refinement. *R*-factors based on *F*^2^ are statistically about twice as large as those based on *F*, and *R*- factors based on ALL data will be even larger.

### Fractional atomic coordinates and isotropic or equivalent isotropic displacement parameters (Å^2^)

**Table:**

	*x*	*y*	*z*	*U*_iso_*/*U*_eq_	Occ. (<1)
Cd1	0.56424 (5)	0.130201 (10)	0.55013 (3)	0.02848 (13)	
O1	0.7075 (5)	0.13446 (11)	0.7651 (3)	0.0416 (9)	
OW1	0.8374 (4)	0.09822 (9)	0.5298 (3)	0.0312 (7)	
HW1A	0.8890	0.1109	0.4809	0.037*	
HW1B	0.9250	0.0942	0.5979	0.037*	
O2	0.5183 (5)	0.07925 (12)	0.7288 (3)	0.0456 (9)	
OW2	0.2993 (5)	0.16577 (13)	0.5563 (3)	0.0578 (12)	
HW2A	0.2058	0.1667	0.4891	0.069*	
HW2B	0.2668	0.1774	0.6207	0.069*	
O3	0.9536 (5)	0.18494 (9)	1.2167 (3)	0.0336 (8)	
OW3	0.7151 (6)	0.19952 (11)	0.5577 (3)	0.0515 (10)	
HW3A	0.7787	0.2095	0.6338	0.062*	
HW3B	0.7838	0.2084	0.5131	0.062*	
O4	0.9934 (5)	0.13719 (10)	1.3685 (3)	0.0333 (8)	
O5	0.1452 (5)	0.05436 (10)	−0.0081 (3)	0.0349 (8)	
N1	0.4690 (5)	0.14857 (11)	0.3209 (3)	0.0259 (8)	
N2	0.3800 (5)	0.07067 (11)	0.4391 (3)	0.0264 (8)	
C1	0.4799 (7)	0.18203 (14)	0.2489 (5)	0.0333 (11)	
H1	0.5383	0.2064	0.2889	0.040*	
C2	0.4087 (7)	0.18207 (15)	0.1182 (5)	0.0359 (11)	
H2A	0.4202	0.2062	0.0733	0.043*	
C3	0.3204 (7)	0.14672 (15)	0.0531 (4)	0.0316 (10)	
H3	0.2742	0.1462	−0.0347	0.038*	
C4	0.3049 (6)	0.11249 (14)	0.1256 (4)	0.0263 (9)	
C5	0.2193 (6)	0.06945 (14)	0.0947 (4)	0.0287 (10)	
C6	0.2442 (6)	0.04832 (13)	0.2210 (4)	0.0245 (9)	
C7	0.1839 (6)	0.01058 (14)	0.2564 (4)	0.0308 (10)	
H7	0.1212	−0.0094	0.1972	0.037*	
C8	0.2219 (7)	0.00387 (14)	0.3860 (4)	0.0334 (11)	
H8	0.1813	−0.0210	0.4147	0.040*	
C9	0.3191 (7)	0.03368 (14)	0.4727 (4)	0.0312 (10)	
H9	0.3436	0.0278	0.5585	0.037*	
C10	0.3391 (6)	0.07652 (13)	0.3142 (4)	0.0221 (9)	
C11	0.3795 (6)	0.11571 (13)	0.2567 (4)	0.0243 (9)	
C12	0.9333 (6)	0.14869 (14)	1.2546 (4)	0.0251 (9)	
C13	0.8319 (6)	0.11616 (13)	1.1591 (4)	0.0234 (9)	
C14	0.7805 (6)	0.12412 (13)	1.0307 (4)	0.0243 (9)	
H14	0.8081	0.1503	1.0025	0.029*	
C15	0.6886 (6)	0.09410 (14)	0.9428 (4)	0.0274 (10)	
C16	0.6496 (7)	0.05520 (15)	0.9856 (5)	0.0338 (11)	
H16	0.5879	0.0349	0.9274	0.041*	
C17	0.7005 (7)	0.04609 (14)	1.1122 (5)	0.0337 (11)	
H17	0.6737	0.0197	1.1393	0.040*	
C18	0.7922 (6)	0.07636 (14)	1.2003 (4)	0.0285 (10)	
H18	0.8271	0.0702	1.2863	0.034*	
C19	0.6332 (6)	0.10307 (16)	0.8034 (4)	0.0321 (11)	
OW4	0.1300 (5)	0.07422 (14)	0.7339 (3)	0.0625 (12)	
HW4A	0.1331	0.0676	0.8169	0.075*	
HW4B	0.2467	0.0716	0.7319	0.075*	
OW5	0.2756 (7)	0.19371 (15)	0.7778 (4)	0.0766 (14)	
HW5A	0.1698	0.1891	0.7959	0.092*	
HW5B	0.3185	0.2198	0.8040	0.092*	
OW6	0.3635 (6)	0.25918 (11)	0.5216 (3)	0.0536 (11)	
HW6A	0.3927	0.2793	0.5723	0.064*	
HW6B	0.4312	0.2386	0.5545	0.064*	
OW7	0.9155 (6)	0.20440 (13)	0.8111 (3)	0.0589 (11)	
HW7A	0.8662	0.1781	0.8182	0.071*	
HW7B	0.8881	0.2207	0.8634	0.071*	
OW8	0.4647 (6)	0.26328 (12)	0.9142 (4)	0.0279 (9)	0.75
HW8A	0.4693	0.2841	0.8685	0.033*	0.75
HW8B	0.5802	0.2546	0.9405	0.033*	0.75
OW8'	0.477 (2)	0.2319 (4)	1.0368 (17)	0.061 (5)	0.25
H8'	0.4158	0.2104	1.0082	0.073*	0.25
H8''	0.5963	0.2359	1.0493	0.073*	0.25

### Atomic displacement parameters (Å^2^)

**Table:**

	*U*^11^	*U*^22^	*U*^33^	*U*^12^	*U*^13^	*U*^23^
Cd1	0.0292 (2)	0.03009 (19)	0.02268 (19)	0.00057 (15)	0.00189 (14)	−0.00493 (14)
O1	0.044 (2)	0.054 (2)	0.0225 (17)	−0.0072 (17)	0.0024 (16)	−0.0039 (15)
OW1	0.0296 (17)	0.0403 (18)	0.0240 (16)	−0.0001 (14)	0.0079 (14)	0.0083 (14)
O2	0.036 (2)	0.064 (2)	0.034 (2)	−0.0091 (18)	0.0051 (16)	−0.0191 (18)
OW2	0.034 (2)	0.092 (3)	0.036 (2)	0.028 (2)	−0.0080 (17)	−0.033 (2)
O3	0.044 (2)	0.0315 (17)	0.0209 (16)	−0.0052 (15)	0.0032 (15)	−0.0013 (13)
OW3	0.068 (3)	0.039 (2)	0.035 (2)	−0.0166 (19)	−0.0034 (19)	−0.0003 (16)
O4	0.0363 (19)	0.0418 (19)	0.0182 (16)	−0.0087 (15)	0.0019 (14)	0.0018 (13)
O5	0.0340 (18)	0.0456 (19)	0.0220 (17)	−0.0038 (15)	0.0032 (14)	−0.0081 (15)
N1	0.0227 (19)	0.0261 (18)	0.026 (2)	0.0014 (16)	0.0030 (16)	−0.0035 (16)
N2	0.0241 (19)	0.0296 (19)	0.0224 (19)	0.0020 (15)	0.0016 (16)	−0.0012 (15)
C1	0.033 (3)	0.026 (2)	0.044 (3)	−0.003 (2)	0.015 (2)	−0.002 (2)
C2	0.036 (3)	0.034 (3)	0.038 (3)	0.002 (2)	0.012 (2)	0.010 (2)
C3	0.032 (3)	0.037 (2)	0.027 (2)	0.006 (2)	0.011 (2)	0.005 (2)
C4	0.020 (2)	0.033 (2)	0.023 (2)	0.0041 (19)	0.0023 (18)	0.0000 (19)
C5	0.021 (2)	0.035 (2)	0.031 (3)	0.0055 (19)	0.009 (2)	−0.006 (2)
C6	0.021 (2)	0.030 (2)	0.021 (2)	0.0025 (18)	0.0029 (18)	−0.0022 (17)
C7	0.028 (2)	0.029 (2)	0.032 (3)	−0.0005 (19)	0.003 (2)	−0.007 (2)
C8	0.039 (3)	0.025 (2)	0.035 (3)	−0.003 (2)	0.009 (2)	0.005 (2)
C9	0.035 (3)	0.034 (2)	0.023 (2)	0.004 (2)	0.007 (2)	0.0049 (19)
C10	0.019 (2)	0.025 (2)	0.021 (2)	0.0022 (17)	0.0026 (17)	−0.0035 (17)
C11	0.017 (2)	0.029 (2)	0.027 (2)	0.0039 (18)	0.0060 (18)	0.0015 (18)
C12	0.016 (2)	0.038 (2)	0.022 (2)	0.0006 (19)	0.0060 (18)	−0.0037 (19)
C13	0.018 (2)	0.028 (2)	0.026 (2)	0.0011 (17)	0.0085 (18)	−0.0039 (18)
C14	0.021 (2)	0.028 (2)	0.025 (2)	0.0008 (18)	0.0085 (18)	−0.0035 (18)
C15	0.016 (2)	0.038 (2)	0.027 (2)	0.0002 (19)	0.0053 (18)	−0.006 (2)
C16	0.024 (2)	0.039 (3)	0.039 (3)	−0.007 (2)	0.009 (2)	−0.018 (2)
C17	0.033 (3)	0.030 (2)	0.040 (3)	−0.006 (2)	0.014 (2)	−0.001 (2)
C18	0.024 (2)	0.034 (2)	0.027 (2)	0.0000 (19)	0.0061 (19)	0.0018 (19)
C19	0.020 (2)	0.047 (3)	0.027 (2)	0.008 (2)	0.002 (2)	−0.010 (2)
OW4	0.035 (2)	0.115 (4)	0.034 (2)	−0.009 (2)	0.0048 (18)	0.020 (2)
OW5	0.085 (3)	0.096 (3)	0.061 (3)	−0.034 (3)	0.041 (3)	−0.030 (3)
OW6	0.090 (3)	0.0289 (18)	0.035 (2)	0.0020 (19)	0.008 (2)	0.0007 (15)
OW7	0.068 (3)	0.069 (3)	0.041 (2)	−0.021 (2)	0.018 (2)	−0.0136 (19)
OW8	0.036 (2)	0.0227 (19)	0.025 (2)	0.0051 (17)	0.0096 (19)	0.0136 (16)
OW8'	0.060 (10)	0.038 (8)	0.096 (14)	−0.021 (8)	0.040 (10)	0.007 (8)

### Geometric parameters (Å, °)

**Table:**

Cd1—OW2	2.219 (3)	C6—C10	1.388 (6)
Cd1—OW1	2.264 (3)	C7—C8	1.394 (6)
Cd1—O1	2.302 (3)	C7—H7	0.9300
Cd1—N2	2.431 (4)	C8—C9	1.386 (6)
Cd1—OW3	2.449 (3)	C8—H8	0.9300
Cd1—N1	2.492 (4)	C9—H9	0.9300
Cd1—O2	2.645 (4)	C10—C11	1.467 (6)
O1—C19	1.260 (6)	C12—C13	1.508 (6)
OW1—HW1A	0.8387	C13—C14	1.381 (6)
OW1—HW1B	0.8379	C13—C18	1.404 (6)
O2—C19	1.241 (5)	C14—C15	1.386 (6)
OW2—HW2A	0.8440	C14—H14	0.9300
OW2—HW2B	0.8887	C15—C16	1.383 (7)
O3—C12	1.252 (5)	C15—C19	1.502 (6)
OW3—HW3A	0.8910	C16—C17	1.370 (7)
OW3—HW3B	0.8352	C16—H16	0.9300
O4—C12	1.259 (5)	C17—C18	1.393 (6)
O5—C5	1.206 (5)	C17—H17	0.9300
N1—C11	1.323 (6)	C18—H18	0.9300
N1—C1	1.346 (6)	OW4—HW4A	0.9335
N2—C10	1.336 (5)	OW4—HW4B	0.8417
N2—C9	1.345 (6)	OW5—HW5A	0.8459
C1—C2	1.385 (7)	OW5—HW5B	0.9059
C1—H1	0.9300	OW6—HW6A	0.8383
C2—C3	1.387 (7)	OW6—HW6B	0.8348
C2—H2A	0.9300	OW7—HW7A	0.9227
C3—C4	1.376 (6)	OW7—HW7B	0.8391
C3—H3	0.9300	OW8—OW8'	1.663 (16)
C4—C11	1.394 (6)	OW8—HW8A	0.8407
C4—C5	1.501 (6)	OW8—HW8B	0.8376
C5—C6	1.511 (6)	OW8'—H8'	0.8260
C6—C7	1.372 (6)	OW8'—H8''	0.8296
			
OW2—Cd1—OW1	174.63 (14)	C7—C6—C10	118.9 (4)
OW2—Cd1—O1	93.95 (13)	C7—C6—C5	133.6 (4)
OW1—Cd1—O1	89.31 (12)	C10—C6—C5	107.4 (4)
OW2—Cd1—N2	94.20 (13)	C6—C7—C8	116.2 (4)
OW1—Cd1—N2	87.35 (11)	C6—C7—H7	121.9
O1—Cd1—N2	125.57 (12)	C8—C7—H7	121.9
OW2—Cd1—OW3	84.62 (15)	C9—C8—C7	121.0 (4)
OW1—Cd1—OW3	91.66 (12)	C9—C8—H8	119.5
O1—Cd1—OW3	81.41 (12)	C7—C8—H8	119.5
N2—Cd1—OW3	152.96 (12)	N2—C9—C8	123.2 (4)
OW2—Cd1—N1	85.60 (13)	N2—C9—H9	118.4
OW1—Cd1—N1	89.90 (11)	C8—C9—H9	118.4
O1—Cd1—N1	160.53 (12)	N2—C10—C6	126.2 (4)
N2—Cd1—N1	73.81 (12)	N2—C10—C11	123.5 (4)
OW3—Cd1—N1	79.17 (12)	C6—C10—C11	110.2 (4)
OW2—Cd1—O2	90.23 (14)	N1—C11—C4	126.6 (4)
OW1—Cd1—O2	95.13 (11)	N1—C11—C10	124.6 (4)
O1—Cd1—O2	51.81 (11)	C4—C11—C10	108.8 (4)
N2—Cd1—O2	74.44 (11)	O3—C12—O4	124.3 (4)
OW3—Cd1—O2	132.49 (11)	O3—C12—C13	118.7 (4)
N1—Cd1—O2	147.56 (11)	O4—C12—C13	117.1 (4)
C19—O1—Cd1	101.1 (3)	C14—C13—C18	118.7 (4)
Cd1—OW1—HW1A	113.1	C14—C13—C12	121.6 (4)
Cd1—OW1—HW1B	114.9	C18—C13—C12	119.7 (4)
HW1A—OW1—HW1B	107.4	C13—C14—C15	121.6 (4)
C19—O2—Cd1	85.4 (3)	C13—C14—H14	119.2
Cd1—OW2—HW2A	117.1	C15—C14—H14	119.2
Cd1—OW2—HW2B	131.0	C16—C15—C14	118.8 (4)
HW2A—OW2—HW2B	111.6	C16—C15—C19	120.0 (4)
Cd1—OW3—HW3A	117.0	C14—C15—C19	121.2 (4)
Cd1—OW3—HW3B	127.6	C17—C16—C15	121.1 (4)
HW3A—OW3—HW3B	102.0	C17—C16—H16	119.4
C11—N1—C1	114.3 (4)	C15—C16—H16	119.4
C11—N1—Cd1	107.9 (3)	C16—C17—C18	120.0 (4)
C1—N1—Cd1	137.8 (3)	C16—C17—H17	120.0
C10—N2—C9	114.5 (4)	C18—C17—H17	120.0
C10—N2—Cd1	109.7 (3)	C17—C18—C13	119.9 (4)
C9—N2—Cd1	135.7 (3)	C17—C18—H18	120.1
N1—C1—C2	123.3 (4)	C13—C18—H18	120.1
N1—C1—H1	118.3	O2—C19—O1	121.7 (4)
C2—C1—H1	118.3	O2—C19—C15	119.6 (5)
C1—C2—C3	121.0 (4)	O1—C19—C15	118.8 (4)
C1—C2—H2A	119.5	HW4A—OW4—HW4B	104.8
C3—C2—H2A	119.5	HW5A—OW5—HW5B	109.1
C4—C3—C2	116.4 (4)	HW6A—OW6—HW6B	108.2
C4—C3—H3	121.8	HW7A—OW7—HW7B	109.6
C2—C3—H3	121.8	OW8'—OW8—HW8A	163.9
C3—C4—C11	118.3 (4)	OW8'—OW8—HW8B	72.6
C3—C4—C5	133.5 (4)	HW8A—OW8—HW8B	105.4
C11—C4—C5	108.2 (4)	OW8—OW8'—H8'	106.8
O5—C5—C4	128.2 (4)	OW8—OW8'—H8''	81.8
O5—C5—C6	126.4 (4)	H8'—OW8'—H8''	126.5
C4—C5—C6	105.4 (4)		
			
OW2—Cd1—O1—C19	86.5 (3)	C4—C5—C6—C10	−1.2 (4)
OW1—Cd1—O1—C19	−97.7 (3)	C10—C6—C7—C8	0.8 (6)
N2—Cd1—O1—C19	−11.5 (3)	C5—C6—C7—C8	−174.3 (4)
OW3—Cd1—O1—C19	170.5 (3)	C6—C7—C8—C9	−1.4 (7)
N1—Cd1—O1—C19	174.5 (3)	C10—N2—C9—C8	0.0 (6)
O2—Cd1—O1—C19	−0.7 (3)	Cd1—N2—C9—C8	−176.4 (3)
OW2—Cd1—O2—C19	−94.2 (3)	C7—C8—C9—N2	1.0 (7)
OW1—Cd1—O2—C19	85.7 (3)	C9—N2—C10—C6	−0.6 (6)
O1—Cd1—O2—C19	0.7 (3)	Cd1—N2—C10—C6	176.7 (3)
N2—Cd1—O2—C19	171.5 (3)	C9—N2—C10—C11	175.5 (4)
OW3—Cd1—O2—C19	−11.3 (3)	Cd1—N2—C10—C11	−7.2 (5)
N1—Cd1—O2—C19	−176.3 (3)	C7—C6—C10—N2	0.2 (7)
OW2—Cd1—N1—C11	−99.8 (3)	C5—C6—C10—N2	176.5 (4)
OW1—Cd1—N1—C11	83.2 (3)	C7—C6—C10—C11	−176.3 (4)
O1—Cd1—N1—C11	170.8 (3)	C5—C6—C10—C11	0.0 (5)
N2—Cd1—N1—C11	−4.1 (3)	C1—N1—C11—C4	2.4 (6)
OW3—Cd1—N1—C11	174.9 (3)	Cd1—N1—C11—C4	−179.1 (4)
O2—Cd1—N1—C11	−16.3 (4)	C1—N1—C11—C10	−176.2 (4)
OW2—Cd1—N1—C1	78.2 (4)	Cd1—N1—C11—C10	2.3 (5)
OW1—Cd1—N1—C1	−98.9 (4)	C3—C4—C11—N1	−1.3 (7)
O1—Cd1—N1—C1	−11.2 (7)	C5—C4—C11—N1	179.0 (4)
N2—Cd1—N1—C1	173.9 (5)	C3—C4—C11—C10	177.5 (4)
OW3—Cd1—N1—C1	−7.2 (4)	C5—C4—C11—C10	−2.2 (5)
O2—Cd1—N1—C1	161.7 (4)	N2—C10—C11—N1	3.6 (7)
OW2—Cd1—N2—C10	89.9 (3)	C6—C10—C11—N1	−179.7 (4)
OW1—Cd1—N2—C10	−85.0 (3)	N2—C10—C11—C4	−175.2 (4)
O1—Cd1—N2—C10	−172.2 (3)	C6—C10—C11—C4	1.4 (5)
OW3—Cd1—N2—C10	3.5 (5)	O3—C12—C13—C14	7.0 (6)
N1—Cd1—N2—C10	5.7 (3)	O4—C12—C13—C14	−172.5 (4)
O2—Cd1—N2—C10	179.0 (3)	O3—C12—C13—C18	−174.5 (4)
OW2—Cd1—N2—C9	−93.6 (4)	O4—C12—C13—C18	5.9 (6)
OW1—Cd1—N2—C9	91.6 (4)	C18—C13—C14—C15	1.0 (6)
O1—Cd1—N2—C9	4.3 (5)	C12—C13—C14—C15	179.5 (4)
OW3—Cd1—N2—C9	−180.0 (4)	C13—C14—C15—C16	−0.6 (6)
N1—Cd1—N2—C9	−177.8 (4)	C13—C14—C15—C19	180.0 (4)
O2—Cd1—N2—C9	−4.5 (4)	C14—C15—C16—C17	−0.1 (7)
C11—N1—C1—C2	−1.6 (6)	C19—C15—C16—C17	179.3 (4)
Cd1—N1—C1—C2	−179.5 (3)	C15—C16—C17—C18	0.3 (7)
N1—C1—C2—C3	−0.1 (8)	C16—C17—C18—C13	0.1 (7)
C1—C2—C3—C4	1.2 (7)	C14—C13—C18—C17	−0.8 (6)
C2—C3—C4—C11	−0.6 (6)	C12—C13—C18—C17	−179.3 (4)
C2—C3—C4—C5	178.9 (5)	Cd1—O2—C19—O1	−1.1 (4)
C3—C4—C5—O5	2.2 (8)	Cd1—O2—C19—C15	179.9 (4)
C11—C4—C5—O5	−178.2 (4)	Cd1—O1—C19—O2	1.3 (5)
C3—C4—C5—C6	−177.5 (5)	Cd1—O1—C19—C15	−179.7 (3)
C11—C4—C5—C6	2.1 (5)	C16—C15—C19—O2	14.8 (6)
O5—C5—C6—C7	−5.5 (8)	C14—C15—C19—O2	−165.8 (4)
C4—C5—C6—C7	174.2 (5)	C16—C15—C19—O1	−164.2 (4)
O5—C5—C6—C10	179.0 (4)	C14—C15—C19—O1	15.2 (6)

### Hydrogen-bond geometry (Å, °)

**Table:**

*D*—H···*A*	*D*—H	H···*A*	*D*···*A*	*D*—H···*A*
OW1—HW1A···O4^i^	0.84	1.82	2.660 (4)	177
OW1—HW1B···OW4^ii^	0.84	1.88	2.705 (5)	168
OW2—HW2A···O4^iii^	0.84	1.95	2.700 (5)	148
OW3—HW3B···OW8^iv^	0.84	2.11	2.945 (6)	174
OW3—HW3A···OW7	0.89	1.93	2.756 (5)	154
OW5—HW5A···OW7^v^	0.85	1.93	2.714 (6)	154
OW4—HW4B···O2	0.84	1.96	2.787 (5)	167
OW6—HW6A···O3^vi^	0.84	1.90	2.725 (5)	166
OW7—HW7B···OW6^vii^	0.84	1.91	2.715 (5)	159
OW8—HW8A···O3^vi^	0.84	1.92	2.717 (5)	158
OW8—HW8A···O4^vi^	0.84	2.52	3.230 (5)	143
OW8—HW8B···OW6^vii^	0.84	2.01	2.839 (6)	172
OW6—HW6B···OW3	0.83	2.37	3.079 (5)	144
OW6—HW6B···OW2	0.83	2.51	3.055 (6)	124
OW7—HW7A···O1	0.92	1.78	2.646 (5)	155

Symmetry codes: (i) *x*, *y*, *z*−1; (ii) *x*+1, *y*, *z*; (iii) *x*−1, *y*, *z*−1; (iv) *x*+1/2, −*y*+1/2, *z*−1/2; (v) *x*−1, *y*, *z*; (vi) *x*−1/2, −*y*+1/2, *z*−1/2; (vii) *x*+1/2, −*y*+1/2, *z*+1/2.
